# Yield of Low Serum Vitamin B12 Results in General Pediatric Outpatient Practice: Age-Specific Patterns and Associations with Testing Indications and Recorded Clinical Features

**DOI:** 10.3390/diagnostics16203331

**Published:** 2026-10-14

**Authors:** Timucin Imdadoglu, Emel Altuncu, Elif Betul Keskin Cetinkaya, Asli Ozturk Yeniay, Elif Pinar Ayyildiz, Sirin Guven

**Affiliations:** 1 Department of Pediatrics, Sancaktepe Şehit Prof. Dr. İlhan Varank Training and Research Hospital, İstanbul 34785, Turkey; ebkeskin@hotmail.com (E.B.K.C.); dr.asliyeniay@gmail.com (A.O.Y.); ayyildizepinar@gmail.com (E.P.A.); 2 Department of Pediatrics, Division of Neonatology, University of Health Sciences, Hamidiye International School of Medicine, Sancaktepe Şehit Prof. Dr. İlhan Varank Training and Research Hospital, İstanbul 34785, Turkey; emelkayrak@yahoo.com; 3 Department of Pediatrics, University of Health Sciences, Sancaktepe Şehit Prof. Dr. İlhan Varank Training and Research Hospital, İstanbul 34785, Turkey; sirin2006@gmail.com

**Keywords:** vitamin B12, cobalamin, low-result yield, laboratory utilization, diagnostic stewardship, pediatric outpatient practice, age-specific testing

## Abstract

**Background/Objectives:** Serum vitamin B12 testing is frequently ordered in pediatric outpatient practice, either routinely or incidentally as part of broad laboratory panels or in response to symptoms and nutritional concerns; however, how the yield of low serum vitamin B12 results varies across age groups and testing indications remains poorly defined. The primary focus was age-related variation in low serum vitamin B12 results, with recorded testing indications examined as a secondary question and symptom-specific analyses considered exploratory. **Methods:** This single-center retrospective study analyzed testing episodes from children aged 0–18 years attending general pediatric outpatient clinics in 2025. Outcomes were <200 pg/mL (primary) and <300 pg/mL (secondary). Two blinded physicians classified testing indications. Cluster-robust regression evaluated age, sex, and indication; 18 symptom indicators were evaluated in age- and sex-adjusted models with Benjamini–Hochberg correction. **Results:** Of 34,744 episodes, 34,721 were valid; 5.8% were <200 pg/mL and 27.5% were <300 pg/mL. Low-result yield (<200 pg/mL) showed a bimodal age pattern: 7.9% at ages 0–2 years, 2.3% at 3–5 years, and 10.6% at 15–18 years. The number of tests per low result ranged from 9.4 at ages 15–18 years to 42.8 at ages 3–5 years. Routine or incidental testing comprised 63.9% of episodes; crude yield by indication ranged from 4.9% to 7.3%. Compared with 6–11 years, adjusted odds were higher at 0–2 years (aOR 2.81, 95% CI 2.44–3.24) and 15–18 years (aOR 3.92, 95% CI 3.42–4.51). No indication independently predicted the primary outcome; no symptom remained significant for that outcome after false discovery rate correction. **Conclusions:** The yield of low serum vitamin B12 results was strongly age-dependent and bimodal, with higher-yield periods during the first two years of life and adolescence. Broad retrospectively recorded testing indications were not independently associated with results < 200 pg/mL after adjustment for age and sex. These findings support age-informed assessment alongside clinician-directed testing.

## 1. Introduction

Vitamin B12 (cobalamin) is an essential micronutrient required for DNA synthesis, myelination, and one-carbon metabolism. Deficiency during childhood may cause megaloblastic anemia, neurologic impairment, and neurodevelopmental delay; when diagnosis is delayed, neurologic consequences may persist despite subsequent treatment [1,2]. Two developmental periods warrant particular attention. During infancy, vitamin B12 status is strongly influenced by maternal stores and early feeding practices [3,4,5], and deficiency may present with developmental regression or movement disorders even in the absence of anemia [1,5]. During adolescence, increased nutritional demands associated with growth, together with restrictive or plant-based dietary patterns that limit animal-source foods, may increase the risk of inadequate cobalamin status [6,7]. These distinct age-related vulnerabilities underscore the importance of early recognition while also making the clinical assessment of vitamin B12 status across childhood inherently complex.

Serum vitamin B12 measurement is frequently incorporated into pediatric outpatient evaluations, either in response to nonspecific complaints and nutritional concerns or as part of broader laboratory panels [8,9]. Despite this widespread use, the clinical value of current testing practices remains uncertain. Total serum vitamin B12 is an imperfect surrogate for intracellular cobalamin status, and a low concentration does not necessarily establish functional deficiency when interpreted in isolation [10,11]. Moreover, much of the available pediatric literature has examined selected clinical populations, including children with clinically apparent vitamin B12 deficiency or anemia [1,2], or has focused on restricted developmental periods such as infancy, preschool age, or school-age children and adolescents [5,12,13]. Consequently, real-world testing patterns and the yield of low serum vitamin B12 results across the full pediatric age spectrum remain insufficiently characterized.

Against this background, we conducted a large retrospective analysis of real-world serum vitamin B12 testing across the full 0–18-year age range in general pediatric outpatient practice. Our primary focus was age-related variation in the yield of low serum vitamin B12 results. Associations with recorded testing indications were examined as a secondary question, while individual symptom indicators were evaluated in exploratory analyses.

## 2. Materials and Methods

### 2.1. Study Design and Setting

We conducted a single-center retrospective laboratory-utilization study at Sancaktepe Şehit Prof. Dr. İlhan Varank Training and Research Hospital, İstanbul, Türkiye, covering the period from 1 January to 31 December 2025. Serum vitamin B12 testing data were obtained from the hospital laboratory information system. Demographic characteristics and clinical documentation relevant to testing indications and symptom indicators were retrieved from the electronic health records and linked to the corresponding testing episode. The study was designed to evaluate the yield of low serum vitamin B12 results, test-ordering patterns, and associated factors in a tested general pediatric outpatient population rather than to estimate population prevalence.

### 2.2. Study Population and Unit of Analysis

Eligible records comprised serum vitamin B12 testing episodes ordered for children and adolescents aged 0–18 years attending general pediatric outpatient clinics during the study period. Episodes originating from inpatient wards, emergency department visits, pediatric subspecialty clinics, or other outpatient services were excluded.

The testing episode, rather than the individual patient, was the primary unit of analysis. Repeated tests from the same patient were retained as separate episodes to reflect the full outpatient testing workload. Episodes with a valid serum vitamin B12 result were included in biochemical outcome analyses. Episodes without an available result were retained in descriptions of testing volume and sex distribution but excluded from outcome denominators.

### 2.3. Laboratory Measurement

Serum vitamin B12 concentrations were measured by electrochemiluminescence immunoassay (ECLIA) on a cobas e 801 analyzer (Roche Diagnostics, Mannheim, Germany). Results were reported in pg/mL. Manufacturer-provided pediatric reference intervals were used for routine laboratory reporting; however, study outcomes were classified according to the prespecified biochemical thresholds described below. Internal quality control was performed daily at two concentration levels, and external quality assessment was conducted monthly through the KBUDEK national proficiency-testing program (Klinik Biyokimya Uzmanları Derneği İktisadi İşletmesi, İstanbul, Türkiye). Methylmalonic acid and quantitative hematologic parameters, including hemoglobin and mean corpuscular volume, were not included in the study dataset and were therefore not analyzed.

### 2.4. Outcome Definitions, Age Groups, and Low-Result Yield

The primary outcome was a serum vitamin B12 concentration < 200 pg/mL, used as a prespecified low-result threshold [11,14]. The secondary outcome was a serum vitamin B12 concentration < 300 pg/mL, prespecified as a broader low-or-borderline biochemical category encompassing both concentrations < 200 pg/mL and the commonly used borderline interval of 200–299 pg/mL [14,15]. Given the uncertainty surrounding age-specific pediatric cutoffs, this threshold was analyzed as a secondary biochemical outcome rather than as a definitive diagnosis of vitamin B12 deficiency. Serum vitamin B12 < 148 pg/mL was examined descriptively as an additional very-low biochemical threshold previously used in pediatric epidemiologic research [16]. These thresholds were used as operational biochemical categories and were not considered sufficient, in isolation, to establish symptomatic or functional vitamin B12 deficiency.

Age was categorized a priori into five clinically interpretable age groups: 0–2 years, 3–5 years, 6–11 years, 12–14 years, and 15–18 years. These groups were informed by pediatric vitamin B12 studies distinguishing infancy and early childhood, preschool, school-age, and adolescent subgroups [10,17], together with the Bright Futures developmental framework, which distinguishes early adolescence (11–14 years) from subsequent adolescent stages beginning at 15 years [18]. For each prespecified threshold, the yield of low serum vitamin B12 results was calculated as the proportion of valid testing episodes with a result below that threshold. Its reciprocal was reported as the number of tests per low result. These measures describe laboratory results in the tested population; they do not establish functional deficiency or quantify clinical benefit.

### 2.5. Clinical Indication and Symptom Classification

The clinical indication for each testing episode was determined through structured review of the electronic medical record documentation available at the time serum vitamin B12 measurement was ordered. Each episode was assigned to one of four mutually exclusive final indication categories:

Routine or incidental testing: vitamin B12 measurement requested during a well-child visit, general health assessment, or broad laboratory evaluation, with no documented symptom, nutritional risk, previous vitamin B12 abnormality, or disease-monitoring context identified as having prompted the vitamin B12 request.
Clinically indicated testing: testing prompted by symptoms, clinical findings, nutritional or growth concerns, hematologic or neurologic findings, or gastrointestinal or malabsorptive risk factors potentially related to vitamin B12 deficiency.
Vitamin B12 follow-up: repeat testing after a previously documented low or borderline vitamin B12 result or during supplementation or post-treatment monitoring.
Other disease follow-up: vitamin B12 measurement requested during the scheduled monitoring of a documented chronic disease, medication exposure, or other clinical condition, when the primary purpose of the encounter was follow-up of that condition rather than evaluation of vitamin B12 deficiency.

When vitamin B12 was included in a broad laboratory panel during follow-up of a documented condition not primarily related to vitamin B12 deficiency, the episode was classified as other disease follow-up rather than routine or incidental testing. The routine or incidental category described the documented or inferred testing context and did not imply that testing was clinically unnecessary.

The presence of a symptom or clinical finding in the medical record was not, by itself, sufficient to classify an episode as clinically indicated testing. The documented clinical context had to indicate that the symptom, finding, or risk factor plausibly contributed to the decision to request serum vitamin B12 measurement.

To evaluate whether specific presenting features provided predictive information beyond the broad indication categories, potentially relevant symptoms and clinical findings were coded separately using a predefined 18-item framework comprising codes S01–S18. More than one symptom indicator could be assigned to the same testing episode. Symptom coding was performed independently of the overall indication classification. Complete code definitions and representative clinical descriptions are provided in Supplementary Table S1.

### 2.6. Classification Procedure and Inter-Rater Reliability

Before final eligibility filtering, candidate records were independently reviewed by two physician reviewers using the predefined coding guide. Serum vitamin B12 results were removed from the reviewer datasets; therefore, both reviewers were blinded to the biochemical outcome during classification. Records with insufficient or ambiguous documentation were initially labeled uncertain/unclassifiable and kept separate from routine or incidental testing. Initial inter-rater agreement was assessed using exact agreement and Cohen’s κ, including the uncertain/unclassifiable category.

Subsequent classification involved structured reconciliation, senior adjudication, and staged automated text-rule and score-based assignment, as applicable. Overlapping indications were assigned a single final category using the applicable coding-rule priorities, category scores, and adjudication decisions. At the final scoring stage, records without a category-specific signal received a default routine or incidental assignment flagged as low confidence. The classification pathway was recorded for each episode.

### 2.7. Descriptive Statistical Analysis

Categorical variables were summarized as frequencies and percentages. Serum vitamin B12 concentrations were summarized by age group and overall as medians and 25th–75th percentiles (Q1–Q3). For a more detailed age-specific description, serum vitamin B12 concentrations were additionally summarized within single-year age categories using medians, 25th–75th and 10th–90th percentiles, together with the proportions of valid results < 200 and <300 pg/mL. Gaussian kernel smoothing (σ = 1.5 years) was applied separately to each age-specific summary series for descriptive visualization. Individual serum vitamin B12 results were displayed with horizontal jitter to reduce overlap. Within the 0–2-year group, low-result proportions were additionally summarized for infants aged <1 year and children aged 1–2 years. In an additional descriptive analysis, the proportion of results from 200 to <300 pg/mL was calculated within each age group. The yield of low serum vitamin B12 results was calculated overall and stratified by age group and testing indication category, using testing episodes with a valid serum vitamin B12 result as the denominator. Its reciprocal—defined as the average number of tests required to identify one result below the corresponding biochemical threshold—was calculated overall and separately by age group and testing indication category. An additional descriptive scenario quantified the change in testing volume and detection of results < 200 pg/mL if routine or incidental testing at ages 3–11 years were omitted, assuming that all other testing remained unchanged. Overall testing volume was expressed as the number of eligible serum vitamin B12 testing episodes per 100 general pediatric outpatient visits, using the available annual aggregate visit count. Because all eligible testing episodes during the study period were included, no a priori sample size calculation was performed.

### 2.8. Regression Analysis and Statistical Inference

Binary logistic regression models were fitted separately for the primary outcome of serum vitamin B12 < 200 pg/mL and the secondary outcome of serum vitamin B12 < 300 pg/mL. Base models included age group and sex, whereas full models additionally included testing indication category. Children aged 6–11 years, male sex, and routine or incidental testing were used as the reference categories. Associations are reported as adjusted odds ratios (aORs) with 95% confidence intervals (CIs). Patient-level cluster-robust sandwich standard errors were used for the all-episode and vitamin B12 follow-up-excluded analyses. Conventional standard errors were used for the first-test analysis, in which each patient contributed one observation. All covariates were entered as main effects; no interaction terms were included in the regression models. Model discrimination was assessed using the area under the receiver operating characteristic curve (AUC). Within each analysis sample, the incremental contribution of recorded testing indication was summarized as the difference in AUC between the model including age group, sex, and testing indication and the model including age group and sex alone.

In exploratory analyses, associations between each symptom indicator and the two biochemical outcomes were evaluated in separate logistic regression models adjusted for age group and sex. For symptom-specific analyses, the Benjamini–Hochberg false discovery rate procedure was applied separately to the 18 *p* values generated for each biochemical outcome: serum vitamin B12 < 200 pg/mL and <300 pg/mL. As a sensitivity analysis, the age- and sex-adjusted models were repeated after restricting the dataset to the first recorded serum vitamin B12 testing episode for each patient. A separate sensitivity analysis excluded episodes classified as vitamin B12 follow-up. All tests were two-sided, and *p* < 0.05 was considered statistically significant unless otherwise specified.

All statistical analyses were performed using Python (version 3.11; Python Software Foundation, Beaverton, OR, USA). Data management and descriptive statistics were conducted using pandas (version 2.1) and NumPy (version 1.26). Binary logistic regression models with patient-level cluster-robust standard errors were fitted using statsmodels (version 0.14). The Benjamini–Hochberg false discovery rate correction was implemented using the scipy.stats module (SciPy version 1.11). Inter-rater agreement (Cohen’s κ) was calculated using scikit-learn (version 1.3).

## 3. Results

### 3.1. Study Cohort

Of 34,886 candidate records, 142 were excluded during final eligibility filtering, leaving 34,744 eligible serum vitamin B12 testing episodes. Of these, 34,721 had a valid serum vitamin B12 result and formed the denominator for low-result proportions; the 23 episodes without an available result were retained in the descriptions of testing volume and sex distribution. Among all eligible episodes, 18,271 (52.6%) were recorded for female patients and 16,473 (47.4%) for male patients. The 34,721 valid testing episodes represented 32,085 unique patients. Among these patients, 29,620 (92.3%) contributed one valid testing episode and 2465 (7.7%) contributed more than one.

The annual activity summary recorded 175,129 general pediatric outpatient visits in 2025. The 34,744 eligible vitamin B12 testing episodes corresponded to 19.8 testing episodes per 100 visits. Age-stratified outpatient visit counts were unavailable; therefore, age-specific testing rates could not be calculated.

The age distribution of the 34,721 episodes with valid results was as follows: 5968 (17.2%) at ages 0–2 years, 5951 (17.1%) at ages 3–5 years, 12,372 (35.6%) at ages 6–11 years, 5413 (15.6%) at ages 12–14 years, and 5017 (14.4%) at ages 15–18 years. Overall, serum vitamin B12 concentrations were <148 pg/mL in 483 episodes (1.4%), <200 pg/mL in 1998 episodes (5.8%), and <300 pg/mL in 9542 episodes (27.5%).

For the candidate records reviewed before final eligibility filtering, initial exact agreement between the two reviewers was 93.6%, with a Cohen’s κ of 0.899. Following structured reconciliation, 641 records with unresolved or uncertain classifications required final adjudication by the senior pediatrician.

### 3.2. Age-Specific Yield of Low Results

The yield of low serum vitamin B12 results differed substantially across age groups (Table 1 and Figure 1). For the primary outcome of serum vitamin B12 < 200 pg/mL, the proportions were 7.9% at ages 0–2 years, 2.3% at 3–5 years, 3.0% at 6–11 years, 9.0% at 12–14 years, and 10.6% at 15–18 years.

The corresponding number of tests required to identify one serum vitamin B12 result below the 200 pg/mL threshold was 12.6, 42.8, 33.7, 11.2, and 9.4 across the five age groups, respectively.

The secondary outcome of serum vitamin B12 < 300 pg/mL showed a similar age-related pattern, with yields of 26.1%, 16.3%, 21.2%, 39.8%, and 44.5% across the five age groups, respectively. The corresponding numbers of tests required to identify one serum vitamin B12 result below the 300 pg/mL threshold were 3.8, 6.1, 4.7, 2.5, and 2.2.

Median serum vitamin B12 concentrations were 405 pg/mL at ages 0–2 years and 394 pg/mL at ages 6–11 years, compared with 328 and 314 pg/mL at ages 12–14 and 15–18 years, respectively (Table 1). The proportions of results from 200 to <300 pg/mL were 18.20% and 18.22% at ages 0–2 and 6–11 years, respectively, compared with 30.87% and 33.90% in the two adolescent groups.

Figure 2 presents individual serum vitamin B12 results and single-year age summaries with smoothed descriptive curves for ages 0–17 years. In infants younger than 1 year, the observed median was 373 pg/mL (interquartile range, 255–544; 10th–90th percentiles, 182–737), compared with medians of 421–442 pg/mL at ages 3–6 years. At ages 14–17 years, medians were 311.5–318 pg/mL, and both the lower and upper quartiles were lower than at ages 3–6 years. The observed proportions of results < 200 and <300 pg/mL were 13.4% and 35.4%, respectively, in infants younger than 1 year, 1.5% and 15.0% at age 6, and 12.6% and 45.9% at age 16. Within the 0–2-year group, the corresponding proportions among children aged 1–2 years were 5.7% (244/4259) and 22.4% (954/4259), respectively.

### 3.3. Low-Result Yield by Testing Indication

Among testing episodes with a valid serum vitamin B12 result, routine or incidental testing accounted for 63.9% of episodes, followed by clinically indicated testing (29.0%), vitamin B12 follow-up (4.6%), and other disease follow-up (2.6%) (Table 2).

Crude low-result yield for serum vitamin B12 < 200 pg/mL ranged from 4.9% in other disease follow-up to 7.3% in vitamin B12 follow-up. The corresponding range for serum vitamin B12 < 300 pg/mL was 25.3% to 33.0%, with vitamin B12 follow-up showing the highest yield across both thresholds. Variation in crude yield across indication categories was substantially narrower than that observed across age groups (Table 2).

To illustrate the potential scope of age-informed test selection, we evaluated a hypothetical scenario in which routine or incidental serum vitamin B12 testing was omitted at ages 3–11 years, the age range with the lowest yield. With all other testing unchanged, 84.1% of the observed results < 200 pg/mL (1680/1998) would still be detected, while testing volume would decrease by 35.0% (12,149/34,721 episodes). This would correspond to 38.2 tests omitted per low result not detected.

### 3.4. Adjusted Associations with Low Vitamin B12

In models adjusted for age group, sex, and testing indication, age was strongly associated with both biochemical outcomes (Table 3). Compared with children aged 6–11 years, the adjusted odds of serum vitamin B12 < 200 pg/mL were higher at ages 0–2 years (aOR 2.81, 95% CI 2.44–3.24), 12–14 years (aOR 3.23, 95% CI 2.81–3.72), and 15–18 years (aOR 3.92, 95% CI 3.42–4.51), but lower at ages 3–5 years (aOR 0.78, 95% CI 0.64–0.95). Clinically indicated testing was not independently associated with serum vitamin B12 < 200 pg/mL (aOR 1.03, 95% CI 0.93–1.14; *p* = 0.574). For serum vitamin B12 < 300 pg/mL, the same age-related pattern was observed, with adjusted odds ranging from 0.73 at ages 3–5 years to 3.02 at ages 15–18 years relative to the 6–11-year reference group. Vitamin B12 follow-up was not independently associated with the primary outcome of serum vitamin B12 < 200 pg/mL (aOR 1.07, 95% CI 0.87–1.31; *p* = 0.513). For the secondary outcome of serum vitamin B12 < 300 pg/mL, however, vitamin B12 follow-up was associated with modestly higher adjusted odds relative to routine or incidental testing (aOR 1.12, 95% CI 1.00–1.26; *p* = 0.048). Neither clinically indicated testing nor other disease follow-up was independently associated with the secondary outcome. Female sex was associated with lower adjusted odds of both serum vitamin B12 < 200 pg/mL (aOR 0.90, 95% CI 0.82–0.98; *p* = 0.021) and serum vitamin B12 < 300 pg/mL (aOR 0.86, 95% CI 0.81–0.90; *p* < 0.001).

In the first-test analysis, 32,085 patients contributed one observation each, with 1897 results < 200 pg/mL and 8912 results < 300 pg/mL. For the primary < 200 pg/mL outcome, the adjusted odds ratios for ages 0–2 and 15–18 years were 2.84 (95% CI, 2.46–3.28) and 4.12 (3.58–4.74), respectively, relative to ages 6–11 years. For <300 pg/mL, the corresponding estimates were 1.34 (1.24–1.44) and 3.16 (2.94–3.40). The first-test estimates, 95% confidence intervals, and *p* values for all four non-reference age groups and female sex are presented in Supplementary Table S3.

After exclusion of 1593 vitamin B12 follow-up episodes, 33,128 valid episodes remained, including 1881 results < 200 pg/mL and 9016 results < 300 pg/mL. The adjusted odds ratios for ages 0–2 and 15–18 years were 2.91 (95% CI, 2.52–3.37) and 4.07 (3.52–4.69), respectively, for <200 pg/mL, and 1.31 (1.22–1.42) and 3.13 (2.91–3.37) for <300 pg/mL. These age contrasts were consistent in direction and approximate magnitude with the main findings (Supplementary Table S3).

Adding recorded testing indication to the age- and sex-based model produced small numerical increases in AUC. In the all-episode analysis, AUC values were 0.6629 versus 0.6647 for results < 200 pg/mL and 0.6381 versus 0.6401 for results < 300 pg/mL. In the first-test analysis, the corresponding values were 0.6676 versus 0.6686 and 0.6406 versus 0.6423, respectively. All AUC increases were <0.003 (Supplementary Table S3).

### 3.5. Exploratory Symptom-Specific Analyses

After adjustment for age and sex and correction for multiple comparisons, no individual symptom indicator was independently associated with serum vitamin B12 < 200 pg/mL (Supplementary Table S2). For the secondary outcome of serum vitamin B12 < 300 pg/mL, hair loss (aOR 1.29, 95% CI 1.08–1.53; FDR-adjusted *p* = 0.036) and weight loss or failure to gain weight (aOR 1.50, 95% CI 1.18–1.90; FDR-adjusted *p* = 0.015) remained significantly associated with higher adjusted odds.

## 4. Discussion

### 4.1. Principal Findings and Routine Testing Practice

In this large retrospective analysis of serum vitamin B12 testing in general pediatric outpatient practice, low-result yield was shaped predominantly by age rather than by the recorded reason for testing. Serum vitamin B12 concentrations < 200 pg/mL were identified in 5.8% of valid testing episodes, but yield varied more than fourfold across age groups, being higher among children aged 0–2 years and adolescents and lowest during the preschool and school-age periods. Nearly two-thirds of tests were ordered routinely or incidentally. Nevertheless, crude yield varied only within a relatively narrow range across indication categories. Broad testing indication categories were not independently associated with serum vitamin B12 < 200 pg/mL after multivariable adjustment, and no individual symptom indicator remained significant after adjustment for age and sex and false discovery rate correction. These findings indicate that serum vitamin B12 testing is used broadly in pediatric outpatient practice, whereas age provides the clearest stratification of low-result yield.

### 4.2. Age-Specific Yield Pattern and Comparison with Existing Literature

The bimodal yield pattern identified in this study—higher during the first two years of life and again during adolescence, with a relative nadir during the preschool and school-age periods—is broadly consistent with age-related patterns reported in pediatric studies from Türkiye, India, and Venezuela [2,12,17,19,20]. Yavuz et al. [17] reported clustering of vitamin B12 deficiency in the 0–2- and 12–17-year age groups in a single-center pediatric outpatient cohort. In Northern India, Umasanker et al. [2] also described a bimodal age distribution among children evaluated for anemia, although their selected clinical population differs from our broader outpatient testing cohort. In nationally representative Indian data, Shalini et al. [19] found vitamin B12 deficiency to be substantially more frequent among adolescents (31.0%) than among school-age (17.3%) or preschool children (13.8%), supporting the adolescent component of the pattern observed in our study. In the Vargas State component of a Venezuelan study of socioeconomically vulnerable children, vitamin B12 deficiency was more prevalent in infants and in the adolescent groups studied at ages 11 and 15 years, providing a close qualitative parallel to the two higher-yield periods observed in our cohort [20]. In a prospective Turkish study restricted to children aged 6–72 months, Elgormus et al. [12] reported the highest frequency of deficiency among those aged 6–11 months, supporting the concentration of early-life risk around the complementary-feeding transition.

The early-life high-yield period is biologically plausible given the strong dependence of infant vitamin B12 status on maternal stores, maternal intake during pregnancy and lactation, and early feeding practices [5]. Infants who are exclusively or predominantly breastfed by mothers with low or marginal vitamin B12 status may remain particularly vulnerable until complementary foods provide reliable dietary sources. Population-based studies from Mexico and Nepal similarly identified greater vitamin B12 deficiency in the youngest age groups examined: prevalence was highest at 1 year of age in Mexico, while children aged 6–11 and 12–17 months in Nepal had higher adjusted odds of deficiency than those aged 18–23 months [21,22]. The lower yield in the preschool and school-age groups may partly reflect the establishment of a more varied diet before the increased nutritional demands and behavioral changes associated with puberty. By contrast, the higher yield in adolescents is likely multifactorial and may reflect increased nutritional demands during pubertal growth together with restrictive or plant-based dietary patterns that limit animal-source foods [6,7]. These factors were not directly measured in our study and should therefore be regarded as plausible explanations rather than demonstrated mechanisms.

The early-childhood component of the bimodal low-result pattern was more pronounced at <200 pg/mL and attenuated, but still present, at <300 pg/mL. Compared with children aged 6–11 years, the 0–2-year group had a similar median but a wider interquartile range and a greater proportion of results below 200 pg/mL, whereas the proportions from 200 to <300 pg/mL were nearly identical (Table 1; Section 3.2). Adolescents, in contrast, had lower medians and quartiles, consistent with a general shift toward lower serum vitamin B12 concentrations. These descriptive differences help explain the contrasting threshold sensitivity of the two components, without establishing a shared biological mechanism. The single-year age summaries and their smoothed descriptive curves illustrate these distributional differences in greater detail (Figure 2). Accordingly, part of the observed threshold-based age pattern may reflect age-related distributional differences rather than corresponding differences in clinically confirmed deficiency risk. Female sex was associated with modestly lower adjusted odds of both biochemical outcomes. The direction of this association is consistent with a recent Istanbul study reporting a higher frequency of low vitamin B12 concentrations among boys [13]. However, the sex-related finding should be regarded as secondary and hypothesis-generating because dietary intake, supplementation, pubertal stage, and other potential explanatory factors were not available in our dataset.

### 4.3. Clinical Indications and Symptom-Specific Findings

In secondary analyses, the broad testing indication categories derived retrospectively from clinical records were not independently associated with serum vitamin B12 < 200 pg/mL after adjustment for age and sex. Vitamin B12 follow-up was associated only with modestly higher adjusted odds of the secondary < 300 pg/mL outcome, which may reflect the inclusion of children with previously low or borderline results. The small numerical increases in AUC after adding recorded testing indication, also observed in the first-test analysis, support its limited incremental contribution to discrimination beyond age and sex within the broad categories evaluated. These findings concern the recorded categories rather than the value of individualized clinical assessment.

Nonspecific complaints overlap with common pediatric conditions [8,23], and routine documentation may incompletely capture dietary risk, symptom severity, or the clinician’s reasoning. Restriction to general pediatric outpatient practice may also limit the range of clinical presentations. In addition, total serum vitamin B12 does not correspond perfectly with functional cobalamin status [11,24].

The symptom-specific analyses were exploratory. No individual symptom remained statistically significant for the primary < 200 pg/mL outcome after adjustment for age and sex and false discovery rate correction. Associations of hair loss and weight loss or failure to gain weight with the secondary < 300 pg/mL outcome were modest and may reflect broader nutritional inadequacy or residual age-related confounding rather than vitamin B12-specific clinical signals; prospective confirmation is needed.

### 4.4. Clinical Implications and Future Research

The marked variation in low-result yield across age groups supports the use of age as a risk-stratification variable rather than as a criterion for either automatically ordering or withholding serum vitamin B12 testing. Greater awareness may be warranted during infancy and adolescence, whereas testing during lower-yield periods should remain guided by individual dietary risk, medical history, examination findings, and clinical judgment. Age-informed diagnostic stewardship should therefore refine, rather than replace, clinician-directed testing [9]. At the same time, our findings do not provide sufficient evidence to support universal pediatric screening, routine testing of otherwise unselected children, or routine vitamin B12 supplementation based solely on age. The hypothetical scenario illustrates the trade-off between testing volume and low-result detection, rather than establishing the clinical safety or cost-effectiveness of omitting routine or incidental testing.

Prospective studies should first determine which factors explain the observed age pattern. In early life, this will require integrated assessment of maternal vitamin B12 status, infant feeding, and complementary dietary intake; during adolescence, dietary patterns, supplementation, and pubertal stage should be evaluated together. A second objective should be to determine whether low total serum vitamin B12 concentrations at different ages correspond to clinically meaningful deficiency. This will require age-sensitive validation of total serum vitamin B12 against functional biomarkers such as methylmalonic acid and holotranscobalamin [10,24,25]. Longitudinal studies should additionally evaluate whether these biochemical patterns predict clinically relevant outcomes and response to treatment [1]. Future studies should also evaluate the economic implications of age-informed testing strategies from a public health perspective.

### 4.5. Strengths and Limitations

This study has several important strengths. It included all eligible serum vitamin B12 testing episodes recorded over a complete calendar year in general pediatric outpatient clinics, providing a large real-world cohort spanning the full 0–18-year age range. The sample size enabled stable age-specific estimates and detailed comparisons across developmental periods that are often combined in smaller pediatric studies. Testing indications were classified using a prespecified framework by two physician reviewers blinded to vitamin B12 results, with high initial agreement and structured reconciliation of discordant records. In addition, analyses accounted for repeated testing at the patient level, and restriction to the first recorded episode per patient produced virtually unchanged age-related estimates. Together, these features strengthen confidence that the observed age gradient reflects a consistent pattern in outpatient testing practice rather than an artifact of repeated measurements or unstructured classification.

Several limitations should nevertheless be considered. First, the retrospective, single-center design may limit generalizability to populations with different dietary, socioeconomic, demographic, or test-ordering characteristics. Because only children selected for testing were included, the observed age pattern, particularly the adolescent peak, may partly reflect age-dependent testing propensity (selection by indication), rather than differences in underlying vitamin B12 status alone. Second, clinical indications and symptom indicators were derived from routine medical records and may not fully capture the clinician’s reasoning, symptom severity or duration, dietary history, or prior supplementation. Third, total serum vitamin B12 concentrations were used as operational biochemical outcomes; methylmalonic acid and quantitative hematologic data were not available in the study dataset, and low results therefore cannot be equated with clinically manifest or metabolically confirmed deficiency in every episode. Finally, maternal vitamin B12 status, infant feeding details, dietary intake, pubertal stage, supplementation, subsequent treatment, and clinical outcomes were not available for analysis. These limitations constrain causal and mechanistic interpretation but do not diminish the principal descriptive finding that the yield of low serum vitamin B12 results varied markedly and consistently by age.

## 5. Conclusions

In this large real-world analysis of pediatric outpatient testing, the yield of low serum vitamin B12 results varied markedly by age, with higher-yield periods during the first two years of life and adolescence and substantially lower yield during the preschool and school-age years. In secondary analyses, broad testing indication categories derived retrospectively from clinical records were not independently associated with serum vitamin B12 < 200 pg/mL after adjustment for age and sex. These findings support incorporating age into clinical risk stratification and diagnostic stewardship while preserving individualized testing based on dietary, developmental, and medical risk. Prospective studies using age-sensitive functional biomarkers and clinical outcomes are needed to determine which low serum vitamin B12 results represent clinically meaningful deficiency.

## Figures and Tables

**Figure 1:**
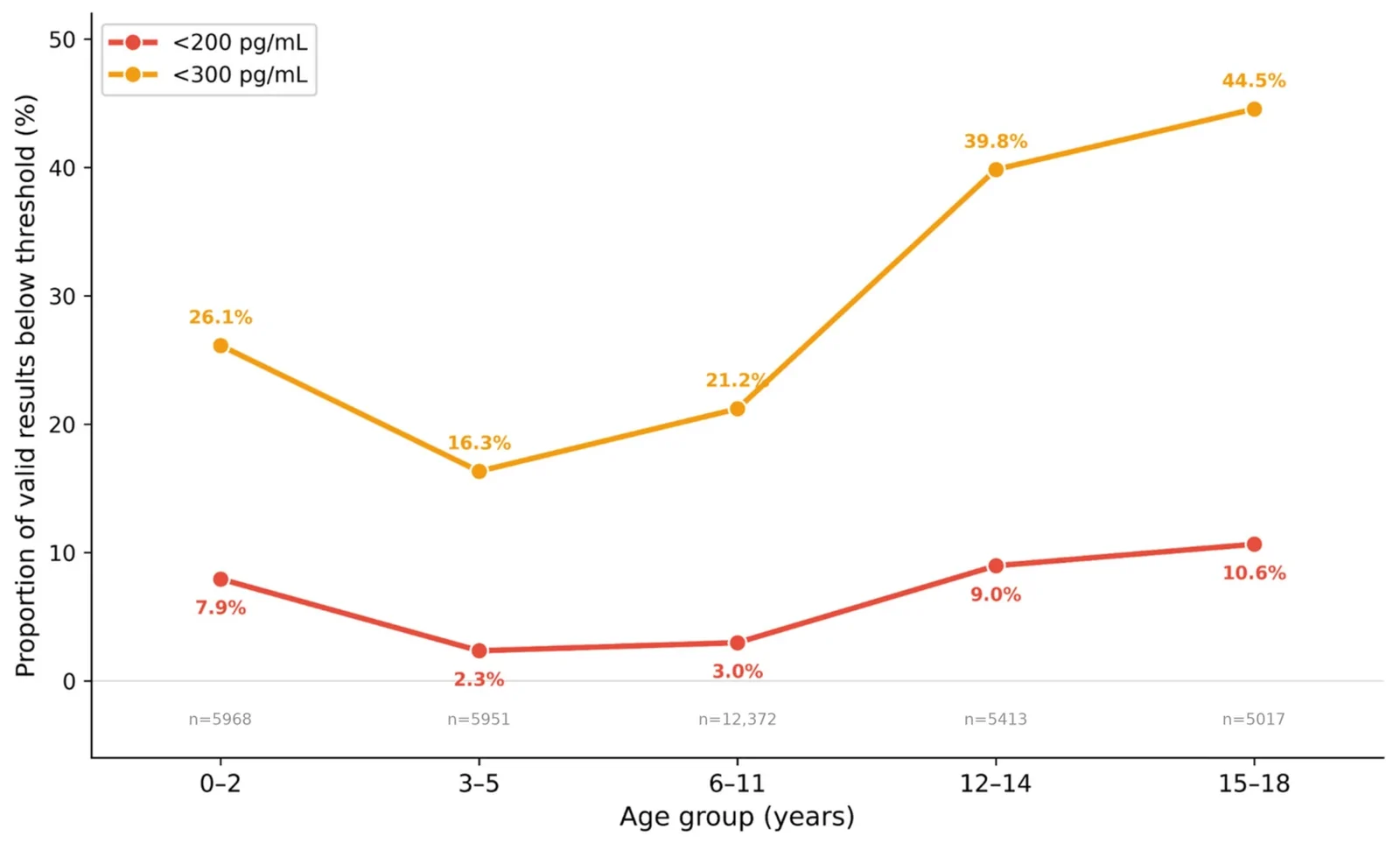
Age-specific proportions of low serum vitamin B12 results in general pediatric outpatients. Data represent 34,721 valid testing episodes from children aged 0–18 years in 2025. Points show the percentages of valid results < 200 and <300 pg/mL within each prespecified age group. Lines connect age-group summaries for visualization. Numbers below the x-axis indicate the number of valid testing episodes in each age group. The corresponding numbers of tests per low result are reported in Table 1.

**Figure 2:**
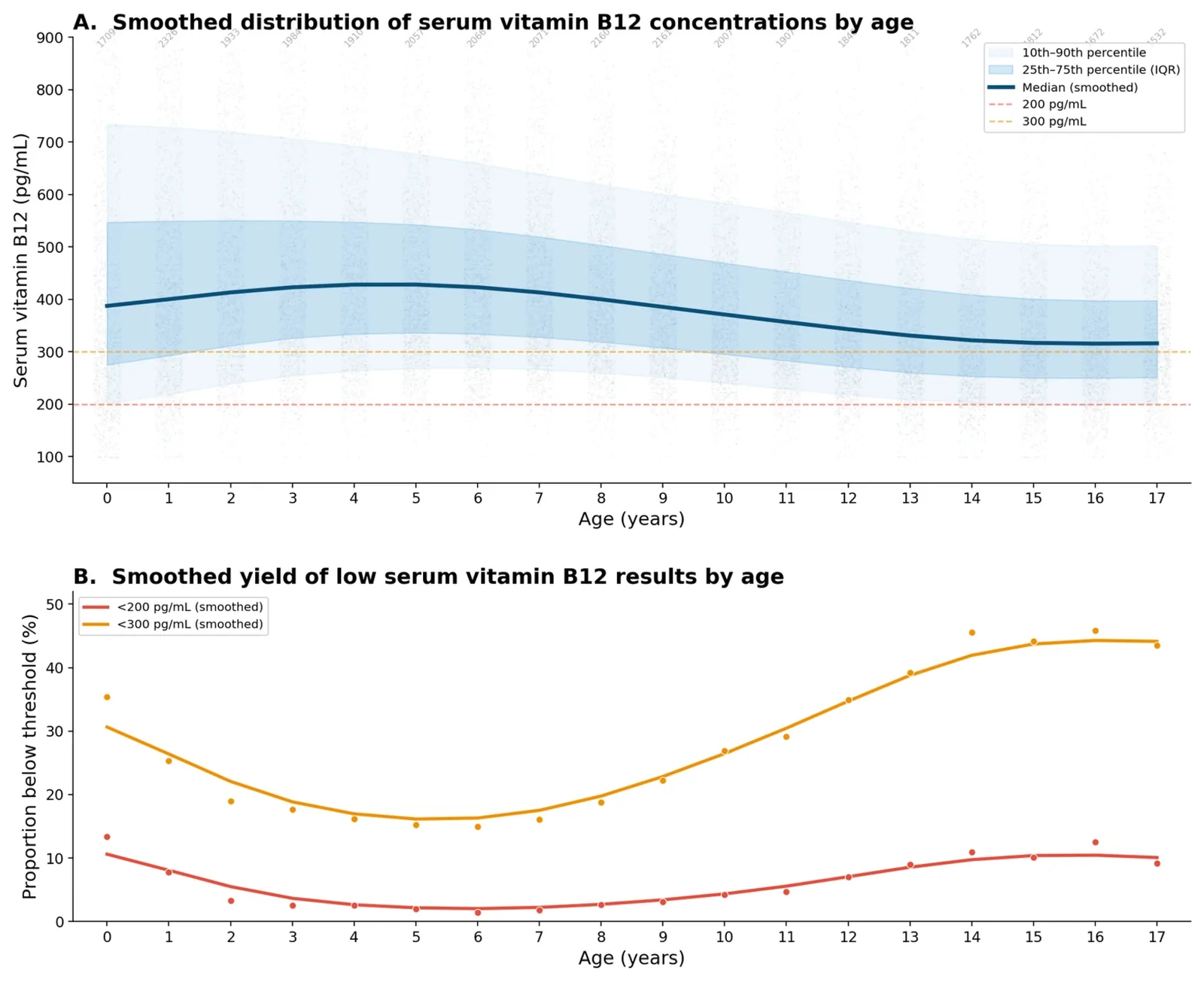
Serum vitamin B12 distributions and low-result proportions by age in general pediatric outpatients, with smoothed descriptive curves. Single-year summaries represent 34,720 valid testing episodes at ages 0–17 years in 2025. Age 0 denotes testing episodes in children younger than 1 year. (**A**) Gray points show individual serum vitamin B12 results with horizontal jitter to reduce overlap. The solid line shows the smoothed median; the darker and lighter shaded bands show the smoothed 25th–75th percentiles (interquartile range, IQR) and 10th–90th percentiles, respectively. These bands describe the distribution of results, not confidence intervals. Dashed horizontal lines mark the study thresholds of 200 and 300 pg/mL. Numbers above Panel (**A**) indicate valid testing-episode counts by age. The displayed y-axis range is 50–900 pg/mL. (**B**) Points show the observed proportions of valid results < 200 and <300 pg/mL within each single-year age category; solid curves show the corresponding smoothed proportions. Gaussian kernel smoothing (σ = 1.5 years) was applied to the single-year median, percentile, and proportion series. The curves are descriptive and do not represent within-child trajectories. The single testing episode at age 18 years was omitted from this figure but retained in the prespecified grouped analyses.

**Table 1:** Serum vitamin B12 concentrations, low-result proportions, and tests per low result by age group in general pediatric outpatients.

Age Group	Valid *n* (%)	Serum B12, Median (Q1–Q3) (pg/mL)	<200 *n* (%)	Tests/ <200	<300 *n* (%)	Tests/ <300
0–2 years	5968 (17.2)	405.0 (294.0–552.0)	473 (7.93)	12.6	1559 (26.12)	3.8
3–5 years	5951 (17.1)	430.0 (337.5–548.0)	139 (2.34)	42.8	971 (16.32)	6.1
6–11 years	12,372 (35.6)	394.0 (313.0–497.3)	367 (2.97)	33.7	2621 (21.18)	4.7
12–14 years	5413 (15.6)	328.0 (257.0–418.0)	485 (8.96)	11.2	2156 (39.83)	2.5
15–18 years	5017 (14.4)	314.0 (250.0–397.0)	534 (10.64)	9.4	2235 (44.55)	2.2
Total	34,721 (100.0)	377.0 (291.0–491.0)	1998 (5.75)	17.4	9542 (27.48)	3.6

*n* = 34,721 valid testing episodes in children aged 0–18 years attending general pediatric outpatient clinics in 2025. Serum vitamin B12 is presented as the median (25th–75th percentile [Q1–Q3]) in pg/mL; threshold values are also in pg/mL. Percentages in the Valid *n* (%) column reflect each age group’s share of all valid episodes; percentages below each threshold use the corresponding age-group denominator. Tests/<200 and Tests/<300 represent the number of tests per result below the respective threshold, calculated as the reciprocal of the low-result proportion. Percentages may not sum to 100% because of rounding.

**Table 2:** Yield of low serum vitamin B12 results and tests per low result by recorded testing indication in general pediatric outpatients.

Testing Indication	Valid *n* (%)	<200 *n* (%)	Tests/<200	<300 *n* (%)	Tests/<300
Routine or incidental	22,184 (63.9)	1225 (5.52)	18.1	5836 (26.31)	3.8
Clinically indicated	10,058 (29.0)	613 (6.09)	16.4	2956 (29.39)	3.4
Vitamin B12 follow-up	1593 (4.6)	117 (7.34)	13.6	526 (33.02)	3.0
Other disease follow-up	886 (2.6)	43 (4.85)	20.6	224 (25.28)	4.0
Total	34,721 (100.0)	1998 (5.75)	17.4	9542 (27.48)	3.6

*n* = 34,721 valid testing episodes in children aged 0–18 years in 2025. Testing indications were classified from clinical documentation into mutually exclusive categories. All thresholds are in pg/mL. Percentages in the Valid *n* (%) column describe the distribution of testing indications; percentages below each threshold use the valid-episode count within the corresponding indication category. Tests/<200 and Tests/<300 are calculated as the valid-episode count divided by the number of results below the respective threshold. Twenty-three episodes with missing results were excluded from outcome denominators (routine or incidental: 17; clinically indicated: 6). Percentages may not sum to 100% because of rounding.

**Table 3:** Adjusted associations of age, sex, and recorded testing indication with serum vitamin B12 results < 200 and <300 pg/mL in general pediatric outpatients.

Variable	<200 pg/mL aOR (95% CI)	*p*-Value	<300 pg/mL aOR (95% CI)	*p*-Value
**Age Group**				
0–2 years	2.81 (2.44–3.24)	<0.001	1.31 (1.22–1.41)	<0.001
3–5 years	0.78 (0.64–0.95)	0.015	0.73 (0.67–0.79)	<0.001
6–11 years	Reference	—	Reference	—
12–14 years	3.23 (2.81–3.72)	<0.001	2.48 (2.31–2.66)	<0.001
15–18 years	3.92 (3.42–4.51)	<0.001	3.02 (2.81–3.24)	<0.001
**Sex**				
Male	Reference	—	Reference	—
Female	0.90 (0.82–0.98)	0.021	0.86 (0.81–0.90)	<0.001
**Testing Indication**				
Routine or incidental	Reference	—	Reference	—
Clinically indicated	1.03 (0.93–1.14)	0.574	1.04 (0.99–1.10)	0.117
Vitamin B12 follow-up	1.07 (0.87–1.31)	0.513	1.12 (1.00–1.26)	0.048
Other disease follow-up	0.82 (0.60–1.12)	0.214	0.88 (0.75–1.03)	0.100

*n* = 34,721 valid testing episodes in children aged 0–18 years in 2025. Separate multivariable logistic regression models were fitted for serum vitamin B12 < 200 and <300 pg/mL, each including age group, sex, and testing indication. Patient-level cluster-robust standard errors accounted for repeated testing. Reference categories were ages 6–11 years, male sex, and routine or incidental testing. aOR, adjusted odds ratio; CI, confidence interval.

## Data Availability

The data presented in this study are available from the corresponding author upon reasonable request, subject to institutional and ethical approval. The data are not publicly available because they contain patient-level clinical information and are subject to institutional data-protection restrictions.

## Supplementary Materials

The following supporting information can be downloaded at: https://www.mdpi.com/article/10.3390/diagnostics16203331/s1, Table S1, Definitions and representative clinical descriptions of the 18 symptom indicators used to code recorded clinical features in pediatric serum vitamin B12 testing episodes; Table S2, Age- and sex-adjusted associations of individual symptom indicators with serum vitamin B12 results < 200 and <300 pg/mL in general pediatric outpatients; Table S3, Selected sensitivity estimates and model discrimination for low serum vitamin B12 results in general pediatric outpatients.
