# Supplementary material for: Yield of Low Serum Vitamin B12 Results in General Pediatric Outpatient Practice: Age-Specific Patterns and Associations with Testing Indications and Recorded Clinical Features
Source: Diagnostics (Basel). 2026 Oct 14;16(20):3331. doi: 10.3390/diagnostics16203331 (PMC13703530; doi:10.3390/diagnostics16203331)
Supplement: Supplementary file 1 [file diagnostics-16-03331-s001.zip › Diagnostics_Supplementary_Tables_S1_S2.pdf]

Supplementary Material

Yield of Low Serum Vitamin B12 Results in General Pediatric Outpatient Practice: Age-Specific Patterns and Associations with Testing Indications and Recorded Clinical Features

Supplementary Table S1. Definitions and representative clinical descriptions of the 18 symptom indicators used to code recorded clinical features in pediatric serum vitamin B12 testing episodes.

Code	Indicator	Representative Clinical Descriptions
S01	Fatigue / Tiredness	Fatigue, tiredness, lack of energy, malaise, or decreased activity levels.
S02	Dizziness	Vertigo, lightheadedness, disequilibrium, or postural dizziness.
S03	Headache	Recurrent or persistent headache, nonspecific head pain, or migraine-like complaints.
S04	Syncope / Fainting	Fainting episodes, transient loss of consciousness, or blackouts.
S05	Numbness / Tingling / Paresthesia	Numbness, tingling, pins-and-needles sensation, or other documented paresthesia.
S06	Memory / Concentration / Attention difficulty	Forgetfulness, memory complaints, difficulty concentrating, or attention problems.
S07	Palpitations	Awareness of heartbeat, rapid or pounding heartbeat, or documented tachycardia.
S08	Hair loss	Excessive hair shedding, thinning of hair, or alopecia-like findings.
S09	Pallor / Anemia suspicion	Pale appearance, conjunctival pallor, or clinical suspicion of anemia.
S10	Loss of appetite	Decreased appetite, picky eating, difficulty feeding (infants), or refusal of foods.
S11	Weight loss or failure to gain weight	Unintentional weight loss, lack of expected weight gain, failure to thrive, or underweight for age.
S12	Growth or developmental delay	Growth faltering or slowed linear growth; delayed motor, language, or cognitive milestones; developmental regression.
S13	Abdominal pain	Diffuse or localized abdominal pain, recurrent abdominal pain, or abdominal cramping.
S14	Nausea / Vomiting	Nausea, vomiting, recurrent vomiting, or episodic emesis.
S15	Constipation	Infrequent or difficult bowel movements, hard stools, painful defecation, or documented functional constipation.
S16	Muscle or leg pain	Myalgia, leg pain, limb pain, or other nonspecific musculoskeletal pain.
S17	Shortness of breath	Dyspnea at rest or with exertion, breathlessness, or difficulty breathing.
S18	Frequent infections	Recurrent upper or lower respiratory tract infections, recurrent otitis media, or other repeatedly documented infections.

More than one symptom indicator could be assigned to the same testing episode. Symptom coding was performed independently of the overall indication classification. Codes S01–S18 constitute the predefined 18-item symptom framework applied to all episodes.

Supplementary Table S2. Age- and sex-adjusted associations of individual symptom indicators with serum vitamin B12 results <200 and <300 pg/mL in general pediatric outpatients.

Panel A. Serum vitamin B12 <200 pg/mL

Code	N	aOR (95% CI)	p	FDR-p
S01	1,074	1.22 (0.96–1.55)	0.098	0.586
S02	842	1.02 (0.78–1.34)	0.866	0.875
S03	277	1.04 (0.66–1.63)	0.875	0.875
S04	694	1.15 (0.87–1.53)	0.336	0.875
S05	109	0.60 (0.26–1.39)	0.234	0.843
S06	362	0.95 (0.62–1.46)	0.807	0.875
S07	303	1.18 (0.78–1.76)	0.434	0.875
S08	653	0.96 (0.69–1.34)	0.800	0.875
S09	281	1.07 (0.68–1.67)	0.784	0.875
S10	239	1.11 (0.65–1.89)	0.714	0.875
S11	326	1.08 (0.68–1.70)	0.754	0.875
S12	134	0.87 (0.38–2.01)	0.751	0.875
S13	124	1.08 (0.52–2.25)	0.834	0.875
S14	359	1.39 (0.96–2.02)	0.080	0.586
S15	84	2.02 (1.02–3.99)	0.043	0.586
S16	35	0.56 (0.08–4.18)	0.575	0.875
S17	58	0.37 (0.09–1.51)	0.165	0.743
S18	27	0.59 (0.08–4.39)	0.604	0.875

Panel B. Serum vitamin B12 <300 pg/mL

Code	N	aOR (95% CI)	p	FDR-p
S01	1,074	1.01 (0.88–1.16)	0.933	0.933
S02	842	1.14 (0.98–1.33)	0.096	0.273
S03	277	1.18 (0.92–1.52)	0.200	0.397
S04	694	1.03 (0.87–1.22)	0.730	0.773
S05	109	0.77 (0.51–1.17)	0.220	0.397
S06	362	1.28 (1.02–1.60)	0.031	0.139
S07	303	1.19 (0.94–1.52)	0.152	0.342
S08	653	1.29 (1.08–1.53)	0.004	0.036*
S09	281	1.11 (0.86–1.44)	0.413	0.619
S10	239	1.09 (0.82–1.46)	0.547	0.703
S11	326	1.50 (1.18–1.90)	0.001	0.015*
S12	134	0.92 (0.61–1.38)	0.669	0.767
S13	124	0.68 (0.45–1.05)	0.083	0.273
S14	359	1.29 (1.03–1.61)	0.027	0.139
S15	84	1.46 (0.92–2.31)	0.106	0.273
S16	35	0.68 (0.29–1.59)	0.376	0.615
S17	58	1.19 (0.70–2.03)	0.518	0.703
S18	27	1.19 (0.52–2.73)	0.681	0.767

*n = 34,721 valid testing episodes. Models adjusted for age group and sex. Benjamini-Hochberg FDR correction applied separately for each outcome across all 18 symptoms. S08 (hair loss) and S11 (weight loss or failure to gain weight) reached FDR-adjusted significance for B12 <300 pg/mL. No symptom reached FDR-adjusted significance for B12 <200 pg/mL. * FDR-significant. aOR: adjusted odds ratio; CI: confidence interval; FDR-p: Benjamini-Hochberg corrected p-value.*
