# Supplementary material for: Yield of Low Serum Vitamin B12 Results in General Pediatric Outpatient Practice: Age-Specific Patterns and Associations with Testing Indications and Recorded Clinical Features
Source: Diagnostics (Basel). 2026 Oct 14;16(20):3331. doi: 10.3390/diagnostics16203331 (PMC13703530; doi:10.3390/diagnostics16203331)
Supplement: Supplementary file 1 [file diagnostics-16-03331-s001.zip › Diagnostics_Supplementary_Table_S3.pdf]

Supplementary Table S3

Yield of Low Serum Vitamin B12 Results in General Pediatric Outpatient Practice: Age-Specific Patterns and Associations with Testing Indications and Recorded Clinical Features

Selected sensitivity estimates and model discrimination for low serum vitamin B12 results in general pediatric outpatients.

A. Base versus extended model discrimination

Base model: age group and sex. Extended model: age group, sex, and recorded testing indication. Models were compared within the same analysis sample.

Analysis	N	Threshold (pg/mL)	Base AUC	Extended AUC
All episodes	34,721	<200	0.6629	0.6647
All episodes	34,721	<300	0.6381	0.6401
First test	32,085	<200	0.6676	0.6686
First test	32,085	<300	0.6406	0.6423

Across these comparisons, adding recorded testing indication produced only small numerical increases in AUC (<0.003).

B. First-test sensitivity analysis

The first-test analysis included one observation per patient (N = 32,085) and used conventional standard errors. Reference categories were ages 6–11 years and male sex.

Threshold (pg/mL)	Patients, n	Results below threshold, n
<200	32,085	1,897
<300	32,085	8,912

Threshold (pg/mL)	Age group / sex	aOR	95% CI	p value
<200	0–2 years	2.8371	2.4572–3.2757	<0.001
<200	3–5 years	0.7815	0.6393–0.9554	0.016
<200	12–14 years	3.2449	2.8117–3.7448	<0.001
<200	15–18 years	4.1161	3.5759–4.7379	<0.001
<200	Female	0.909	0.827–0.999	0.048
<300	0–2 years	1.3382	1.2416–1.4424	<0.001
<300	3–5 years	0.7296	0.6714–0.7928	<0.001
<300	12–14 years	2.5163	2.341–2.7047	<0.001
<300	15–18 years	3.1599	2.935–3.402	<0.001
<300	Female	0.8695	0.8266–0.9146	<0.001

C. Excluding vitamin B12 follow-up episodes

After exclusion of 1,593 episodes classified as vitamin B12 follow-up, 33,128 valid testing episodes remained. This exclusion refers to the recorded follow-up category and does not establish verified supplementation status for every child.

Threshold (pg/mL)	Episodes, n	Results below threshold, n
<200	33,128	1,881
<300	33,128	9,016

Threshold (pg/mL)	Age contrast (reference: 6–11 years)	aOR (95% CI)
<200	0–2 years	2.91 (2.52–3.37)
<200	15–18 years	4.07 (3.52–4.69)
<300	0–2 years	1.31 (1.22–1.42)
<300	15–18 years	3.13 (2.91–3.37)

D. Selected indication estimates in the all-episode extended model

Primary outcome: serum vitamin B12 <200 pg/mL. Reference indication: routine or incidental testing.

Recorded testing indication	aOR (95% CI)	p value
Clinically indicated	1.03 (0.93–1.14)	0.57
Vitamin B12 follow-up	1.07 (0.87–1.31)	0.52
Other disease follow-up	0.82 (0.60–1.12)	0.21

Notes

All-episode and vitamin B12 follow-up-excluded analyses used patient-level cluster-robust sandwich standard errors; the first-test analysis used conventional standard errors. Base models included age group and sex, and extended models additionally included recorded testing indication. No interaction terms were included. AUC, area under the receiver operating characteristic curve; aOR, adjusted odds ratio; CI, confidence interval.
